# Noninvasive Optical Measurement of Cerebral Blood Flow in Mice Using Molecular Dynamics Analysis of Indocyanine Green

**DOI:** 10.1371/journal.pone.0048383

**Published:** 2012-10-31

**Authors:** Taeyun Ku, Chulhee Choi

**Affiliations:** 1 Graduate School of Medical Science and Engineering, KAIST, Daejeon, Republic of Korea; 2 Department of Bio and Brain Engineering, KAIST, Daejeon, Republic of Korea; 3 KI for the BioCentury, KAIST, Daejeon, Republic of Korea; University of Cambridge, United Kingdom

## Abstract

In preclinical studies of ischemic brain disorders, it is crucial to measure cerebral blood flow (CBF); however, this requires radiological techniques with heavy instrumentation or invasive procedures. Here, we propose a noninvasive and easy-to-use optical imaging technique for measuring CBF in experimental small animals. Mice were injected with indocyanine green (ICG) via tail-vein catheterization. Time-series near-infrared fluorescence signals excited by 760 nm light-emitting diodes were imaged overhead by a charge-coupled device coupled with an 830 nm bandpass-filter. We calculated four CBF parameters including arrival time, rising time and mean transit time of a bolus and blood flow index based on time and intensity information of ICG fluorescence dynamics. CBF maps were generated using the parameters to estimate the status of CBF, and they dominantly represented intracerebral blood flows in mice even in the presence of an intact skull and scalp. We demonstrated that this noninvasive optical imaging technique successfully detected reduced local CBF during middle cerebral artery occlusion. We further showed that the proposed method is sufficiently sensitive to detect the differences between CBF status in mice anesthetized with either isoflurane or ketamine–xylazine, and monitor the dynamic changes in CBF after reperfusion during transient middle cerebral artery occlusion. The near-infrared optical imaging of ICG fluorescence combined with a time-series analysis of the molecular dynamics can be a useful noninvasive tool for preclinical studies of brain ischemia.

## Introduction

Measurement of cerebral blood flow (CBF) is essential for the diagnosis and monitoring of stroke patients. Nuclear imaging methods that detect radioactive tracers by positron emission tomography [1] and radiological imaging methods, such as computed tomography (CT) [2], [3] and magnetic resonance imaging (MRI) [4], [5], are widely used for noninvasive measurement of CBF. In basic research involving small animals, the application of these methods is restricted especially for longitudinal studies due to limitations that include the high cost of equipment, risk from radiation, and the low availability of portable devices. As an alternative, optical approaches have gained increasing attention for detection of CBF.

Optical methods for measuring CBF include the use of fluorescent tracers and label-free techniques. Laser Doppler flowmetry, a label-free optical technique, measures the velocity of circulating red blood cells in the bloodstream by the Doppler effect, and is commonly used to monitor the relative change in CBF in experimental animals [6]. Optical micro-angiography, based on optical coherence tomography, was recently developed for the label-free measurement of blood velocity in small vessels in mice [7]; however, practical applications of these techniques in the preclinical setting are limited. Laser Doppler flowmetry is not sensitive enough to estimate regional CBF and detects only the relative changes [8]; whereas optical micro-angiography has a critical limitation in that the scalp of small animals must be removed to obtain sufficient resolution to identify red blood cell movement in small vessels.

In contrast to label-free techniques, optical techniques using fluorescent tracers can significantly enhance the signal-to-noise ratio (SNR) using intravascular contrast agents. In addition, these techniques have the advantage of extracting kinetic information by time-series analysis of fluorescence signals after the bolus injection of the tracers. Indocyanine green (ICG) is a fluorescent probe clinically used for liver function tests [9] and angiography in ophthalmology [10]. ICG is excited in the near-infrared (NIR) wavelength range [11], and deeper penetration of NIR wavelength light in tissues is enabled due to a lesser degree of absorption and scattering [12]. Intravenously injected ICG rapidly binds to serum albumin and remains confined in the vessels until it is removed via clearance through the liver [11]. Kinetic information of the ICG bolus enhances detection of deep organs by enriching of organ-specific information including differences in filling and wash-out patterns [13], [14] and detection of tissue blood flow in experimental animals [15], [16] and humans [17]–[20]. Hillman *et al*. described such approaches in detail and termed them dynamic contrast enhancement (DyCE) technique [21]. We demonstrated recently that analysis of ICG molecular dynamics can be used to estimate peripheral tissue perfusion semi-quantitatively in mice [16] and humans [17], [22]. However, this approach has not been applied for the detection of CBF due to the different hemodynamic characteristics of the brain, which has a rapidly moving and abundant blood supply. Near-infrared spectroscopy (NIRS) has been used primarily to detect ICG absorption signals in the brain [23], [24]. Measurement of CBF using NIRS and ICG was first demonstrated to be effective in animals [15], [25], and was then rapidly applied to humans because of its noninvasive characteristics [18], [26]–[28]. However, detection of NIRS signals above the human scalp is highly limited by the presence of a large amount of noise. This limitation is mainly due to the thick extracerebral tissues, including the scalp, skull, and dura mater; therefore, there have been significant efforts to separate intracerebral from extracerebral signals [27], [29], [30]. While methods such as CT or MRI provide two- (2D) or three-dimensional (3D) images that provide valuable information about CBF, NIRS can detect blood flow information only at several points upon the head due to its limited spatial resolution. It is reported that the detection of ICG fluorescent signal is more sensitive than the detection of absorption signals by NIRS when used as a point detection approach [31], [32]. Woitzik and his colleagues obtained 2D blood flow maps using ICG fluorescent imaging, although the method was applicable only during an invasive surgical procedure where the scalp and skull of the patient were totally removed [33].

While optical penetration is restricted by the thick extracerebral tissues in humans, sufficiently high SNR can be achieved in experimental small animals–such as mice–because of their relatively thin extracerebral tissues. We propose an optical imaging technique that measures CBF in mice noninvasively through a combined ICG molecular dynamics analysis. To the best of our knowledge, this is the first report to examine the feasibility of noninvasive fluorescence imaging for measurement of CBF in small animals.

## Results

### Analysis of the Molecular Dynamics of ICG for Two-dimensional Measurement of CBF in Mice

**Figure 1:**
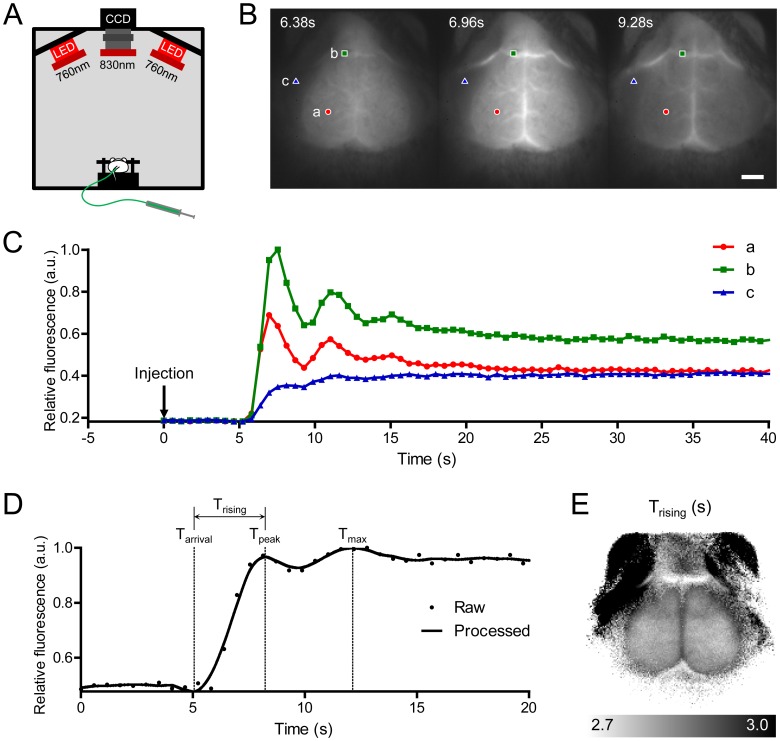
Time-series fluorescence imaging of indocyanine green (ICG) on a mouse head and generation of a cerebral blood flow (CBF) map. (A) Schematic of imaging setup. (B) Time-series acquisition of fluorescence images after intravenous bolus injection of ICG. The scalp was excised and the bregma was positioned at the center of the images (scale bar: 2 mm). (C) ICG dynamics in three pixels after injection of an ICG bolus, indicated in (B). Different regions of interest are indicated by colored shapes (red circle, cerebrum; green rectangle, large vein; blue triangle, skeletal muscle). (D) Smoothed and interpolated dynamics were plotted as a solid line, and the arrival time (T_arrival_) and the first peak time (T_peak_) for calculating the rising time (T_rising_) are indicated on the time axis. (E) A representative CBF map drawn using the T_rising_ parameter. CCD, charge-coupled device; LED, light-emitting diode; a.u., arbitrary unit; T_max_, maximum intensity time.

To obtain the molecular dynamics of ICG in mouse brain, we acquired time-series fluorescence images over the mouse head after a bolus injection of ICG (Figure 1B). The dynamics in each pixel typically consisted of the first peak followed by subsequent peaks formed by systemic recirculation (Figure 1C). The relative intensities and time points of each peak were distinct in different tissues. The time point of the first peak over the cerebrum was earlier than those in the large vein and the extra-cranial skeletal muscle. The first peak in the skeletal muscle showed lower intensity than later peaks. We extracted T_rising_ values from the first peak as a representative parameter of the status of tissue blood supply. T_rising_ was calculated by subtracting T_arrival_, the time of appearance of the bolus, from the first peak time (T_peak_) (Figure 1D). The average T_rising_ value over a somatosensory cortical region of nine mice anesthetized with isoflurane was 2.7±0.1 s. T_rising_ values in each pixel were reconstructed to create a 2D CBF map (Figure 1E). The CBF map clearly revealed the shape of the mouse brain. This finding indicates that ICG fluorescence imaging combined with analysis of molecular dynamics can separate the brain from adjacent tissues using the differences in hemodynamic parameters in mice with intact skulls.

### Intracranial CBF was Dominantly Detected by Time-series Optical Imaging of ICG Fluorescence

**Figure 2:**
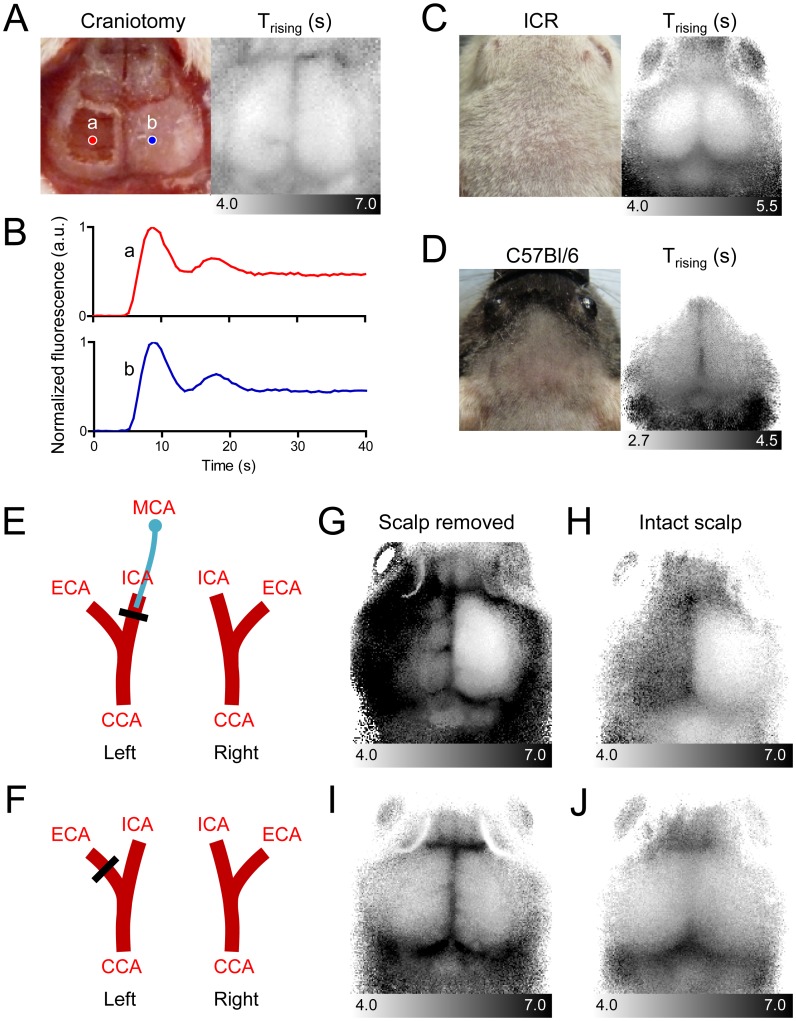
Intracranial blood flow parameters are predominantly measured by dynamic fluorescence imaging analysis. (A) Image of a mouse head (in which the extracerebral tissues were totally removed in the left hemisphere) and a T_rising_ map. To remove extracerebral tissues, the scalp was excised, and a craniotomy was applied along with dura mater excision. (B) ICG dynamics of a region from which all extracerebral tissues were removed (red line) and a contralateral region with intact skull and dura mater (blue line). (C and D) Representative T_rising_ maps of a head with an intact scalp in an ICR mouse (C) and a C57Bl/6 mouse (D). (E and F) Diagrams of the selective block of blood supply to intracerebral (E) and extracerebral (F) tissues. (G and H) Left internal carotid artery (ICA) was ligated, and a nylon suture filament was inserted toward the middle cerebral artery (MCA) to block the intracerebral blood supply. The external carotid artery (ECA) was ligated to block the extracerebral blood supply. T_rising_ maps for the selective block of the intracerebral blood supply in a scalp-removed mouse (G) and an intact-scalp mouse (H). (I and J) T_rising_ maps for the selective block of the extracerebral blood supply in a scalp-removed mouse (I) and an intact-scalp mouse (J). All mice were anesthetized with ketamine and xylazine. CCA, common carotid artery.

We next investigated whether the blood flow information observed in the area of the shape of the mouse cerebrum on the CBF map was originated from intracranial brain tissue. Above the cerebral cortex of the mouse are multiple layers of extracerebral tissues, including the scalp, skull, and dura mater. Because these extracerebral tissues also have blood vessels and blood supply, we compared fluorescence dynamics over the cranium to those directly over the cerebrum. When we removed the extracerebral tissues above the hemisphere of a scalp-removed mouse by craniotomy with complete excision of the dura mater (Figure 2A), we found no difference in the pattern in dynamics between the contralateral region with the skull and dura mater and the ipsilateral region lacking these extracerebral tissues (Figure 2B). We further evaluated whether ICG fluorescence imaging with time-series analysis of molecular dynamics could detect CBF noninvasively. We shaved only the scalp to minimize the invasiveness of the procedure. The CBF maps successfully displayed the shape of the underlying brain both in white ICR mice and black C57Bl/6 mice (Figure 2C and D). Even though the dynamics of the extracerebral flow were not extractable from the dynamics of the intracerebral flow, the degree of influence by the extracerebral signals warranted further evaluation. Therefore, we selectively blocked either the extracerebral or the intracerebral blood flow by selective vascular occlusion, and subsequently examined the CBF maps. To selectively reduce intracerebral blood flow, we ligated the ICA and occluded the MCA by inserting a suture filament that preserved the intact ECA (Figure 2E). To selectively reduce the extracerebral flow, we ligated the ECA (Figure 2F). Selective blockade of the intracerebral blood flow prominently increased the T_rising_ parameters of the pixels in the ipsilateral hemisphere, both in a scalp-removed mouse and in an intact-scalp mouse (Figure 2G and H). In contrast, although ligation of the ECA effectively reduced the delivery of ICG to the extracerebral tissues (Figure S1), it did not significantly alter the T_rising_ map regardless of scalp removal (Figure 2I and J). These results collectively indicate that the time-series optical imaging of ICG fluorescence can successfully detect blood flow, dominantly representing intracerebral blood flow even in the presence of extracerebral tissues in mice.

### Four Types of Blood Flow Map and Application to Cerebral Ischemia

**Figure 3:**
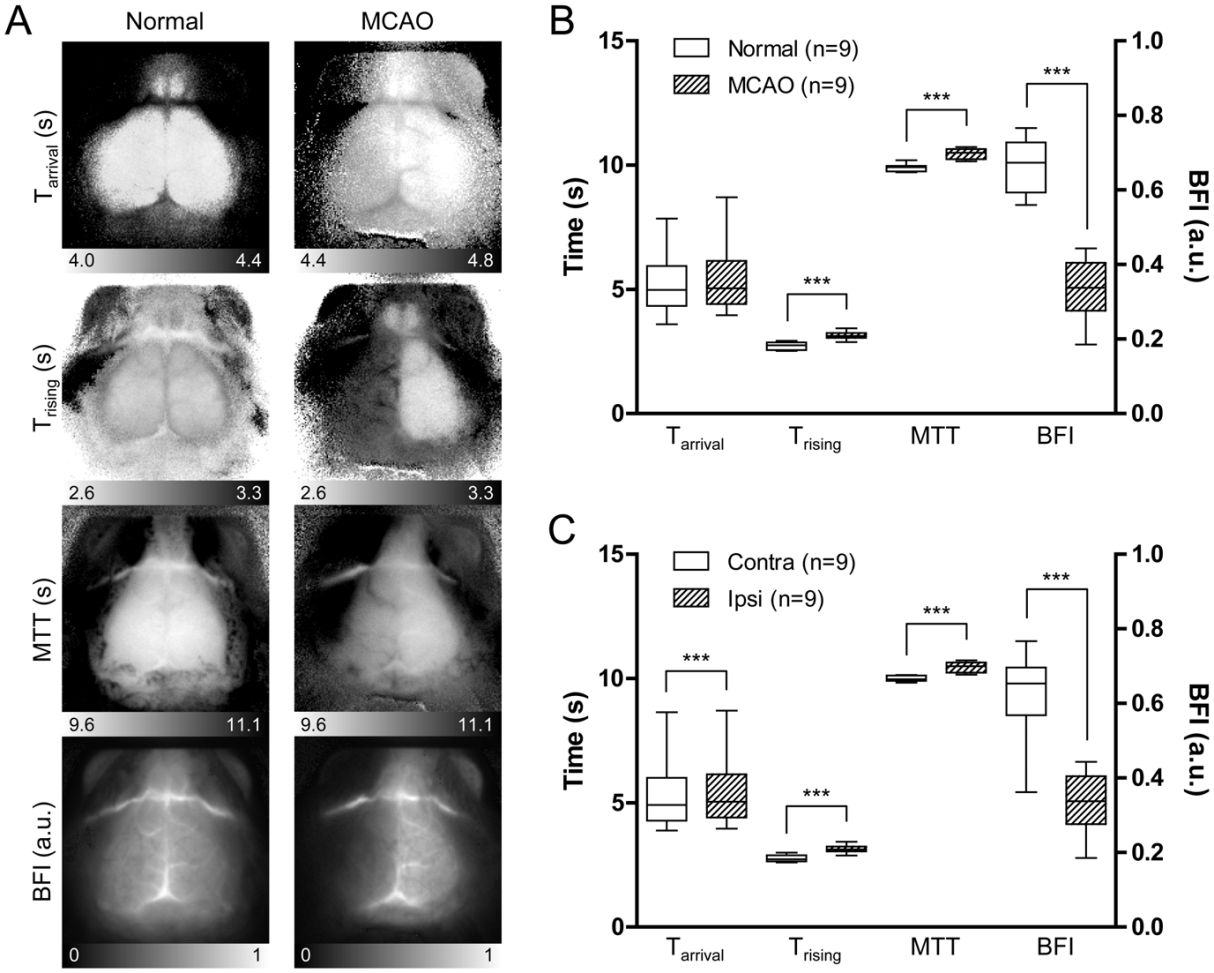
Types of CBF maps and a comparison of CBF parameters in normal and ischemic hemispheres. (A) Representative CBF maps using the four parameters described in Table 1 were generated from a normal condition (left panels) and an ischemic condition in which the left MCA was occluded (right panels). (B) Average blood flow parameters of regions over left somatosensory cortices of nine mice before and after MCA occlusion (MCAO) surgery. T_rising_ and blood flow index (BFI) parameters showed significant differences between the normal and ischemic conditions (*t*-test, ***p<0.001). (C) Average blood flow parameters of regions over ipsilateral ischemic somatosensory cortices were compared to those regions over contralateral normal cortices in nine mice during MCAO. All four parameters showed significant differences (paired *t*-test, ***p<0.001). MTT, mean transit time; Contra, contralateral region; Ipsi, Ipsilateral region.

We next selected three more parameters in addition to T_rising_ from the ICG time kinetics, with or without intensity information, to improve the CBF estimates (Table 1). T_arrival_ was selected as the time axis. We calculated the mean transit time (MTT) as the center of gravity of the dynamic curve and the blood flow index (BFI) as the slope of the first peak [3], [15]. Four parameters were used to generate independent CBF maps. We investigated changes in CBF maps under cerebral ischemia conditions. We induced focal cerebral ischemia in mice by middle cerebral artery occlusion (MCAO) surgery. Representative CBF maps for a normal brain before MCAO and an ischemic brain during MCAO are displayed in Figure 3A. All four maps showed altered hemodynamic parameters in the ischemic hemisphere compared to those in the normal hemisphere. Lateral regions of the cerebral cortex are supplied primarily by the middle cerebral artery (MCA), but they also receive a fraction of their blood supply from the anterior cerebral artery (ACA) on the medial side through anastomoses in the case of MCAO [34]. In ischemic brains where the MCA is occluded, flow from the ACA partially recovers the blood supply of lateral regions. Consistent with this notion, the T_arrival_ map showed a gradual delay in the arrival of the ICG bolus from the medial to the lateral side. However, in the T_rising_ map, the entire cortex in the ipsilateral hemisphere showed increased T_rising_ values. An increase in MTT was also observed in the ipsilateral hemisphere. The dynamic analysis clearly indicated that not only the lateral regions primarily supplied by the MCA but also the medial regions supplied by the ACA received a decreased blood supply by MCAO, although the ACA flow was not disturbed directly. Because the dynamics observed in the supra-capillary resolution dominantly represent capillary flow (Figure S2), the delay in the arrival of the bolus through arterial vessels did not match the tissue blood flow status estimated by T_rising_ and MTT. The BFI values, which utilize the rising time as the denominator, were lower in the lateral regions, indicating a noticeable decrease in blood volume.

**Table 1:** Definitions and interpretations of cerebral blood flow (CBF) map parameters.

Map parameter	Definition	Interpretation	Reference
Arrival time (T_arrival_)	Initiation time of rise of the first passageafter bolus injection.	T_arrival_ reflects the fastest vascular path from the heart.Marked occlusion of major arteries will alter T_arrival_maps. Absolute value of T_arrival_ is affected largely bythe bolus injection method.	[44]
Rising time (T_rising_)*	Time taken to reachthe first peak after arrival of bolus. 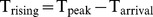	T_rising_ may be a primary choice for evaluation of localtissue perfusion. Volume of blood supply isrepresented in T_rising_ maps with a high contrast.	[26]
Mean transit time (MTT)	Mean detection time of ICGparticles in a region of interest. 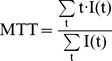	MTT calculates whole blood supply including delayedcollateral flows. MTT may be a better choice in casesof low signal-to-noise ratios.	[27]
Blood flow index (BFI)*	The slope of the first peak ina time-intensity curve. 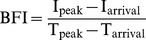	BFI represents overall blood volume informationwith respect to time.	[15], [33]

* Other reports may use T_max_ and I_max_ instead of T_peak_ and I_peak_, respectively.

t, observation time point; I(t), intensity function of time; I_peak_, intensity of the first peak; T_peak_, time of I_peak_; I_arrival_, intensity at T_arrival_; I_max_, maximum intensity; T_max_, time of I_max_.

Average values of parameters in the regions over left somatosensory cortices in nine mice were calculated and compared before and during MCAO (Figure 3B). Because T_arrival_ is affected by the time of initiation of the bolus injection, only T_rising_, MTT, and BFI showed significant differences between groups (*t*-test, *p*<0.001). In contrast, when the parameters in the ipsilateral ischemic hemispheres were compared to those in the contralateral normal hemispheres during MCAO in individual mice, all four parameters exhibited statistically significant differences (paired *t*-test, *p*<0.001) (Figure 3C). These results clearly indicate that CBF parameters and maps are capable of detecting cerebral ischemia status.

**Figure 4:**
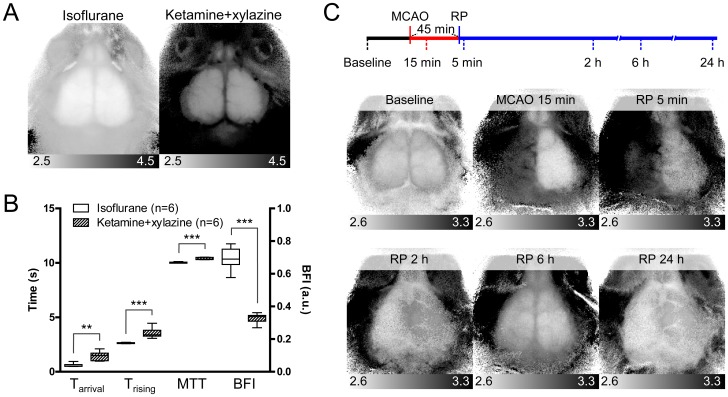
Detection of cerebral hemodynamic changes using CBF maps. (A) Representative T_rising_ maps for mice anesthetized with either 1.5% isoflurane or 0.1 mg/g ketamine and 0.01 mg/g xylazine. (B) Averaged CBF parameters of regions over left somatosensory cortices of six mice anesthetized with isoflurane and six mice anesthetized with ketamine and xylazine. T_arrival_, T_rising_, and MTT parameters increased significantly, and BFI parameter was decreased significantly, in ketamine and xylazine group (*t*-test, **p<0.01; ***p<0.001). (C) Timeline of the transient MCAO protocol (upper diagram) and representative T_rising_ maps (lower panels). Reperfusion (RP) was induced at 45 min after MCA occlusion. The six time points for imaging acquisition are indicated under the timeline.

### Detection of Cerebral Hemodynamic Changes using CBF Maps

We expanded our application of CBF maps to other experimental conditions accompanying cerebral hemodynamic changes. First, we anesthetized two groups of mice using different anesthetics and compared their CBF maps. Inhalation of isoflurane increases CBF, and injection of a ketamine–xylazine cocktail (K–X) causes a decrease in CBF through its effects on systemic hemodynamic functions [35]–[37]. In T_rising_ maps, the overall T_rising_ value in mice anesthetized with K–X was larger than that in mice anesthetized by isoflurane (Figure 4A). Average values in somatosensory regions in the K–X group resulted in significantly increased T_arrival_, T_rising_, and MTT and decreased BFI (*t*-test, *p*  = 0.001 for T_arrival_ and *p*<0.001 for others) (Figure 4B).

Second, we obtained serial CBF maps throughout the time course of transient MCAO (Figure 4C). Images were taken before MCAO surgery, at 15 min after MCAO induction, and at 5 min, and 2, 6, and 24 h after reperfusion following 45 min of occlusion. The CBF status of the two hemispheres could be identified in CBF maps at each imaging time-point. These results indicate that CBF maps can be used to monitor CBF status affected by hemodynamic conditions or CBF changes after cerebral ischemic events.

## Discussion

We demonstrated that optical imaging using the NIR fluorescence dye, ICG, combined with a time-series analysis of the molecular dynamics, can be used for noninvasive analysis of CBF. The patterns of ICG dynamics were distinct in each tissue, and the differences could be successfully expressed using four dynamic parameters including the T_arrival_, T_rising_, MTT, and BFI. Blood flow maps generated using these parameters clearly identified the shape of the mouse cerebrum and detected changes in CBF during cerebral ischemia. Hair removal alone was sufficient for imaging of normal and reduced CBF induced by selective vascular occlusion surgery. We further confirmed that this technique can be used to compare CBF status in animals anesthetized with different anesthetics and to monitor changes in CBF after induction of cerebral ischemia followed by reperfusion.

Our results clearly demonstrate that the dynamics of the intracerebral CBF are dominantly detected by dynamic fluorescence imaging, even in the presence of extracerebral tissues. Due to deeper penetration of NIR wavelength light, signals from sufficiently deep brain tissue are collected, and the portion of detected signals from the relatively thin extracerebral tissues may be negligible. We found that the intensity of fluorescence signals that originated below the scalp was up to four times higher than those from within the scalp. A large portion of extracerebral signals may be masked by strong intracerebral signals, and qualitative analysis of intracerebral flow may not be practically interrupted, as shown in Figure 2H.

Repetitive ICG injections and CBF measurements were feasible as shown in Figure 4C. Reinduction of anesthesia after a recovery period may require repeated catheterization procedures, and the number of attempts for venous puncture on tail veins will be limited. This may restrict the practical repeatability of ICG imaging. During experiments, the injection route was retained using catheterization; waiting 10 min was sufficient to carry out subsequent ICG injections and imaging. However, excessive repetition of injections should be avoided in a single animal over a short period considering plasma volume overload.

Each of the four blood flow parameters provided unique information regarding CBF, and the combined analysis enhanced the interpretation of the CBF condition such as hypoperfusion, anastomoses, and collateral blood supply induced by experimental intervention of CBF. T_arrival_ represents the first detectable bolus to arrive through different passages. Under an ischemic condition, T_arrival_ could be used to indicate which blood supply feeds the ischemic region. Even in the case in which the decrease in tissue blood flow was not so prominent by a T_rising_ map or a MTT map, the T_arrival_ map could be used to determine the success of an attempt to interrupt arterial sources. The T_rising_ parameter provides a highest contrast map between ipsilateral and contralateral hemispheres in MCAO and should be the first choice to estimate changes in CBF status. Because the T_rising_ parameter eliminates the effect of injection time uncertainty by subtracting T_arrival_, a direct comparison of T_rising_ and its derivative, BFI is also possible for results obtained from independent experiments. MTT is traditionally introduced in radiological imaging methods [3] and is currently used for analysis of ICG dynamics detected by methods such as NIRS [27]. MTT is theoretically calculated from the first passage of the bolus [38] but can also be calculated without eliminating recirculation [39]. MTT is referred to as the transition time after the bolus arrives at a pixel or a region of interest, so it is subtracted by arterial input dynamics suitable for the pixel or the region. Practically a large artery observed in the same image is chosen for global use as an arterial input function. As such large reference arteries are hard to utilize in 2D projected fluorescence images, we calculated MTT using fixed interval dynamics after T_arrival_ without compensating for the arterial input function. Although this approach may decrease MTT accuracy compared to that observed in mice using other methods [40], it is sufficient to compare region by region in the same hemisphere or between two hemispheres in an identical mouse. Because a relatively larger amount of information on the time axis is used to calculate MTT than other parameters, the MTT is particularly useful for analyzing low SNR dynamics. BFI is a parameter widely used to analyze ICG dynamics [15], [33]. BFI considers not only temporal information but also intensity information. The intensity value is used for blood volume information during derivation of the BFI [15]. Therefore, the ICG injection volume determines the scale of the BFI, and its control is important for comparisons among animals.

Bolus arrival can be greatly affected by injection method, the size of the animals, and environmental factors such as room temperature. Automatically injecting the ICG bolus using a syringe pump and triggered initiation of imaging acquisition will reduces the variability of bolus arrival and enhance the accuracy of T_arrival_ parameters. T_rising_, MTT, and BFI are free from the injection time variation, and they significantly distinguished the ischemic hemispheres among individual experiments. However, regardless of variation in injection time, all four parameters are good candidates for analyzing blood flow in a single mouse to compare region by region or an ipsilateral hemisphere with a contralateral hemisphere.

We were not only able to detect local remarkable decreases in CBF such as MCAO using CBF maps, but also global CBF changes using different anesthetics or subtle changes in local CBF using serial imaging during the course of transient MCAO (Figure 4). K–X is expected to decrease CBF compared to isoflurane [37], and all four CBF parameters showed corresponding differences. However, users who compare CBF parameters among animals anesthetized with different anesthetics must be aware that MTT can be biased by the anesthetic. Decreased hepatic ICG clearance by isoflurane, in contrast to increased liver enzyme activity by K–X, may reduce the decrease in late-time intensity [41], [42]. Because MTT takes a larger portion of the dynamics into account than do the other parameters, the central gravity of the dynamic might be shifted to the right.

In this study, we used a charge-coupled device (CCD) camera at a low frame rate (580 ms per frame) to enhance the SNR of NIR fluorescence signals and increased the temporal resolution by post-processing of dynamics, including smoothing and interpolation. Signals obtained by high SNR CCD enabled easy and accurate determinations of the initiation points and the maximum points of the first peak rise to calculate T_arrival_ and T_peak_. Other acquisition devices with high frame rates could also be used, and they may provide high temporal resolution. However, fine algorithms to determine T_arrival_ and T_peak_ might be required in the absence of proper signal-processing procedures. Imaging with a higher frame rate system was reported by Hillman *et al*., and alternative mathematical approaches to extraction of different dynamic components from complicated *in vivo* signals were introduced [21]. The technical aspects of tail-vein injection were also described in detail in that study. Despite the non-invasiveness and accessibility advantages, CBF optical imaging by analysis of the molecular dynamics of ICG has limitations in terms of dimensionality. The acquired images were projected in two dimensions, and this method cannot provide 3D blood flow information, as provided by CT or MRI. According to the central volume theorem, the regional CBF can be calculated by dividing the fractional cerebral blood volume by the MTT [3]. However, the reference arterial voxel required to adjust the MTT and to calculate the fraction of blood volume cannot be obtained because the arterial pixel was not identifiable due to poor resolution of the current imaging setting, and the projected pixel contains summated information from all of the vertically aligned non-arterial voxels. Therefore, several hemodynamic parameters, which can be calculated in 3D imaging techniques, may not be applied in this 2D optical imaging method.

Other limitations of the current technique involve quantitative calculation of CBF. First, T_arrival_, T_peak_, T_max_, T_rising_, and MTT are not expressed in terms of volume per unit time per unit tissue weight (*e*.*g*., mL/100 g/min), the standard unit of tissue blood flow. These parameters provide a qualitative rather than quantitative comparison of CBF status. Especially under ischemic conditions, a bolus can arrive from more than two arteries with different time kinetics. Thus, the shape and height of the peaks are altered, making it difficult to estimate the status of CBF using simple parameters. Therefore, more than one parameter should be considered for precise interpretation, and quantitative parameters that do not depend on the shape of the peaks must eventually be devised.

## Materials and Methods

### Animal Preparation

Eight-week-old male ICR and C57Bl/6 mice were obtained from Samtako (Gyeonggi-do, Republic of Korea) and Koatech (Gyeonggi-do, Republic of Korea), respectively. ICR mice were used for all experiments except imaging with intact scalps, which was performed using C57Bl/6 mice. Animals were anesthetized with either 1.5% isoflurane in 70% N_2_O and 30% O_2_ with a vaporizer or ketamine (0.1 mg/g wt) and xylazine (0.01 mg/g wt). Isoflurane was used as a default anesthetic and the use of K-X was indicated in the figure legends. All efforts were made to minimize suffering. Body temperatures were monitored by rectal probe and maintained at 37±1°C using a temperature-controlled heat blanket. The scalp was excised for ordinary ICG imaging, and the scalp hair was shaved when imaging the intact scalp. A 2×2-mm craniotomy was made with a dental drill above the left parieto-temporal cortex and the dura mater was removed to image the cerebral cortex without any extracerebral tissue. The head was fixed in a stereotaxic frame using two ear-bars and a jaw-holder. All procedures were approved by the Institutional Animal Care and Use Committee of the Korea Advanced Institute of Science and Technology (Permit Number: KA2010-39) and performed in accordance with the ARRIVE guidelines (http://www.nc3rs.org/ARRIVE).

### ICG Imaging

Tail veins were catheterized using a 30-gauge needle and polyethylene (PE)-10 tubing. Cardiogreen (I2633; Sigma-Aldrich, St. Louis, MO) was dissolved in phosphate-buffered saline to a concentration of 0.5 g/L. A bolus of 1.5 µL/g wt cardiogreen solution was injected manually through a tail vein catheter after the start of the time-series imaging experiments. Time-series ICG fluorescence signals were acquired using a custom-built device manufactured by Vieworks Corp. (Gyeonggi-do, Republic of Korea). The device was composed of two light-emitting diode arrays emitting light with a peak intensity at 760 nm that passed through a 760±12 nm band-pass filters and a CCD camera with a 25 cm TV lens coupled with an 830±12 nm band-pass filter (Figure 1A). Twelve-bit grayscale images were captured every 580 ms for ∼3 min, and the initial 40 frames were used to generate blood flow maps.

### Analysis of Fluorescence Dynamics

Time-series fluorescence images were analyzed by custom-built graphic-user-interface software using Delphi 2009 (Embarcadero Technologies, San Francisco, CA). ICG dynamics in each pixel were processed to compensate for noise and low temporal resolution (Figure 1D). They were smoothed using the Savitzky–Golay algorithm with third order polynomials and interpolated to a resolution of 11.6 ms per frame using the cubic spline method. The half-intensity time (T_half_) was first identified to find T_arrival_ and T_peak_. T_half_ was defined as the first time at which the intensity was equal or greater than the median intensity. T_half_ was traced backward along the downslope until the intensity did not decrease, and the destination time was designated T_arrival_. In the same manner, T_half_ was traced forward along the upslope until the intensity did not increase, and the destination time was designated T_peak_. T_rising_ was calculated as the difference between T_arrival_ and T_peak_. MTT was calculated as the center of gravity of the dynamic curve [3]. In this study, signals during the 20 s period after T_arrival_ were used in the calculation. After adjusting the baseline intensity to zero, the following equation was applied;
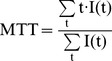
(1)


BFI was defined as the slope of the first peak [15]. The definitions and calculations of the four parameters are summarized in Table 1. Blood flow maps were generated by combining each parameter in each pixel, and the range of the values matching the grayscale intensity was adjusted and displayed under the map in arbitrary units for BFI or in seconds for other parameters. BFI values were normalized for display of each image, but not for comparative analyses among hemispheres. Regions of 2×2 mm in left hemispheres whose centers were positioned 2 mm posterior and 2 mm lateral from the bregma were used to calculate mean blood flow parameters. Symmetric regions in the right hemispheres were compared with the ischemic left hemispheres during MCAO.

### Selective Vascular Occlusion Surgery

MCAO surgery was performed to induce focal cerebral ischemia using a protocol described previously, with some modifications [43]. The neck was incised along the midline, and the left common carotid artery (CCA), the external carotid artery (ECA), and the internal carotid artery (ICA) were exposed. The CCA, ICA, and two positions on the ECA were ligated with 6-0 silk sutures. The ECA was cut partially between two ligated positions, and a 5-0 rounded tip monofilament nylon suture was introduced through the opening in the CCA. The ECA was completely cut, and the suture direction was reversed toward the ICA. We proceeded with the nylon suture for 9 mm from the insertion position. Time-series images were obtained before the surgery and at 15 min after MCAO. The suture was removed at 45 min after its insertion, the ECA was permanently ligated, and the ligations applied to the ICA and the CCA were released for transient MCAO. A series of ICG images was additionally taken at 5 min, and 2, 6, and 24 h after reperfusion. Two positions on the ICA were ligated to selectively block the intracerebral blood supply, and a nylon suture, identical to that used for the MCAO, was inserted between the two positions and proceeded until bifurcation of the MCA. The ECA was ligated to selectively block the extracerebral blood supply.

### Statistical Analysis

Data are expressed as means ± standard errors. Statistical significance was assessed by two-tailed unpaired *t*-tests to compare before and after MCAO surgery and between groups anesthetized with either isoflurane or K–X. The two-tailed paired *t*-test was used to compare the two hemispheres in mice under MCAO.

## Supporting Information

Figure S1 **Reduction of extracerebral flow by occlusion of the ECA.** (A) Under anesthesia with K-X, two round patches that blocked the ICG fluorescence signal were inserted under the scalp through a midline incision, and the left ECA was ligated. (B) The ECA-ligated (a) and control (b) regions are indicated on the BFI map. (C) Fluorescence dynamics of the ECA-ligated and control regions. Note that the increase in fluorescence intensity was attenuated in concordance with the time delay of the maximum intensity in the ECA-ligated region.
(TIF)

Figure S2 **Dynamics of the averaged fluorescence dominantly represents the capillary blood flow in a region of an ischemic cortex.** (A) Under anesthesia with K-X, a fluorescence image was taken above an ischemic cortex of a mouse that received left MCAO surgery during time-series imaging. The images were obtained from a thinned skull window using intravital fluorescence microscopy after a bolus injection of 2 MDa fluorescein isothiocyanate (FITC)-dextran. The arterial branching patterns and their direction indicate that the pial arteries shown are branched from the MCA (scale bar: 200 µm). (B) Fluorescence dynamics of FITC in three arteries (a1, a2, and a3), three regions lacking large vessels (c1, c2, and c3) and three veins (v1, v2, and v3) are indicated. The averaged fluorescence dynamics of a region of interest (white circle) and of ICG in the same region after a bolus injection of ICG were compared.
(TIF)

Methods S1 (DOCX)
