# Supplementary figures and images for: Noninvasive Optical Measurement of Cerebral Blood Flow in Mice Using Molecular Dynamics Analysis of Indocyanine Green
Source: PLoS One. 2012 Oct 31;7(10):e48383. doi: 10.1371/journal.pone.0048383 (PMC3485229; doi:10.1371/journal.pone.0048383)

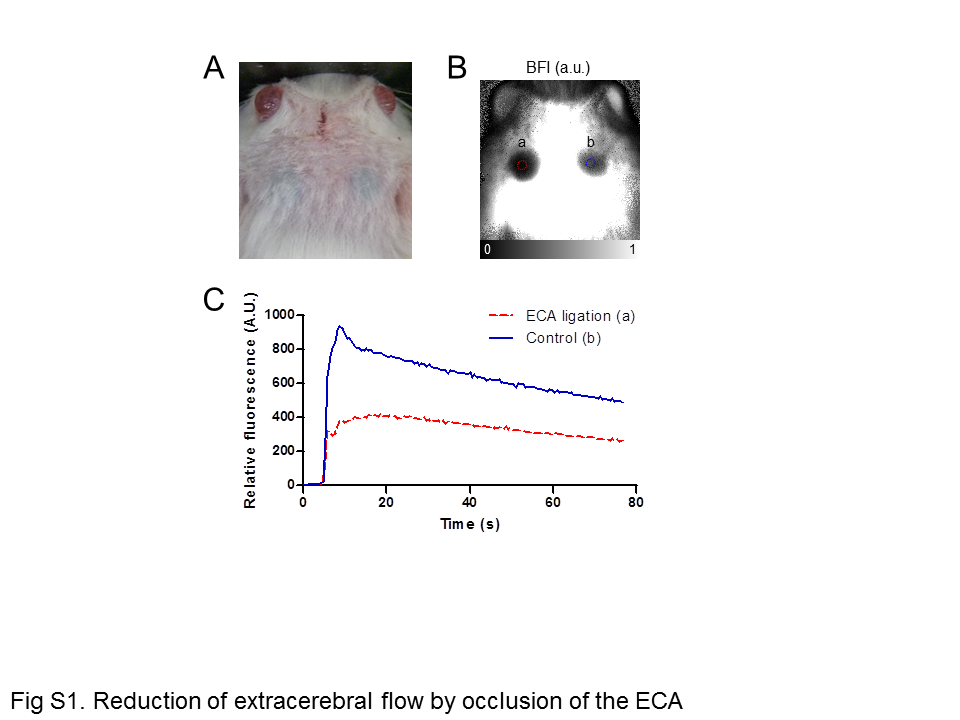

Supplement: Figure S1 — Reduction of extracerebral flow by occlusion of the ECA. (A) Under anesthesia with K-X, two round patches that blocked the ICG fluorescence signal were inserted under the scalp through a midline incision, and the left ECA was ligated. (B) The ECA-ligated (a) and control (b) regions are indicated on the BFI map. (C) Fluorescence dynamics of the ECA-ligated and control regions. Note that the increase in fluorescence intensity was attenuated in concordance with the time delay of the maximum intensity in the ECA-ligated region. (TIF) [file pone.0048383.s001.tif]

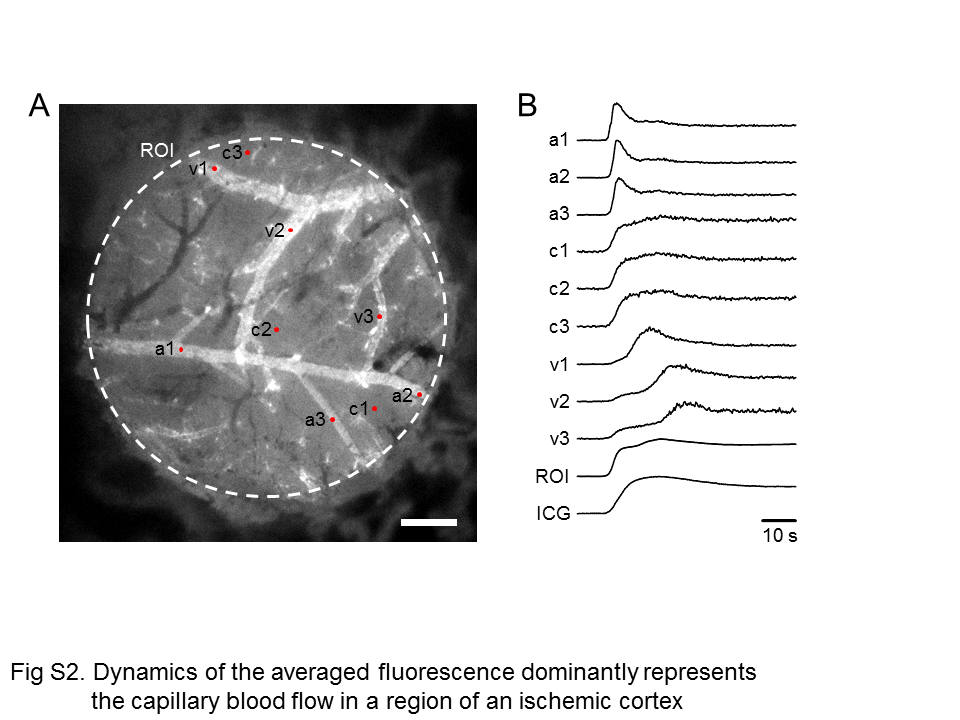

Supplement: Figure S2 — Dynamics of the averaged fluorescence dominantly represents the capillary blood flow in a region of an ischemic cortex. (A) Under anesthesia with K-X, a fluorescence image was taken above an ischemic cortex of a mouse that received left MCAO surgery during time-series imaging. The images were obtained from a thinned skull window using intravital fluorescence microscopy after a bolus injection of 2 MDa fluorescein isothiocyanate (FITC)-dextran. The arterial branching patterns and their direction indicate that the pial arteries shown are branched from the MCA (scale bar: 200 µm). (B) Fluorescence dynamics of FITC in three arteries (a1, a2, and a3), three regions lacking large vessels (c1, c2, and c3) and three veins (v1, v2, and v3) are indicated. The averaged fluorescence dynamics of a region of interest (white circle) and of ICG in the same region after a bolus injection of ICG were compared. (TIF) [file pone.0048383.s002.tif]
