# Supplementary material for: Noninvasive Optical Measurement of Cerebral Blood Flow in Mice Using Molecular Dynamics Analysis of Indocyanine Green
Source: PLoS One. 2012 Oct 31;7(10):e48383. doi: 10.1371/journal.pone.0048383 (PMC3485229; doi:10.1371/journal.pone.0048383)
Supplement: Methods S1 — (DOCX) [file pone.0048383.s003.docx]

**Supplementary Methods**

**Intravital fluorescence microscopy**

High resolution time-series fluorescence imaging was introduced to obtain dynamics of fluorescence signals in individual blood vessels. About 1.5mm-diameter rounded area of the skull over the left somatosensory cortex of a mouse was thinned using a dental drill. 2 MDa fluorescein isothiocyanate (FITC)-dextran dissolved in phosphate-buffered saline to a concentration of 12.5mg/mL was injected intravenously through a tail vein catheter to visualize blood vessels. HBO 100 mercury lamp (Zeiss) was used for illumination source and 4x dry-type objective lens was used. After bolus injection of 2.5μl/g FITC-dextran solution, time-series fluorescence images were taken at a rate of 15 frames per second by a CCD camera (Luca^EM^ S, Andor Technology plc., South Windsor, CT) attached to Zeiss LSM 510 upright microscope system. Every consecutive five images were averaged and each dynamics extracted from pixels in vessels of interest was normalized.
